# Focused Ion Microbeam Irradiation Induces Clustering of DNA Double-Strand Breaks in Heterochromatin Visualized by Nanoscale-Resolution Electron Microscopy

**DOI:** 10.3390/ijms22147638

**Published:** 2021-07-16

**Authors:** Yvonne Lorat, Judith Reindl, Anna Isermann, Christian Rübe, Anna A. Friedl, Claudia E. Rübe

**Affiliations:** 1 Department of Radiation Oncology, Saarland University Hospital, 66421 Homburg, Germany; yvonne.lorat@uks.eu (Y.L.); anna.isermann@uks.eu (A.I.); christian.ruebe@uks.eu (C.R.); 2 Institute for Applied Physic and Metrology, Universität der Bundeswehr München, 85577 Neubiberg, Germany; judith.reindl@unibw.de; 3 Department of Radiation Oncology, University Hospital, Ludwig-Maximilian University, 80539 Munich, Germany; Anna.Friedl@lrz.uni-muenchen.de

**Keywords:** carbon ions, charged-particle radiotherapy, microbeam irradiation, DNA double-strand breaks (DSBs), non-homologous end joining (NHEJ), transmission electron microscopy (TEM)

## Abstract

Background: Charged-particle radiotherapy is an emerging treatment modality for radioresistant tumors. The enhanced effectiveness of high-energy particles (such as heavy ions) has been related to the spatial clustering of DNA lesions due to highly localized energy deposition. Here, DNA damage patterns induced by single and multiple carbon ions were analyzed in the nuclear chromatin environment by different high-resolution microscopy approaches. Material and Methods: Using the heavy-ion microbeam SNAKE, fibroblast monolayers were irradiated with defined numbers of carbon ions (1/10/100 ions per pulse, ipp) focused to micrometer-sized stripes or spots. Radiation-induced lesions were visualized as DNA damage foci (γH2AX, 53BP1) by conventional fluorescence and stimulated emission depletion (STED) microscopy. At micro- and nanoscale level, DNA double-strand breaks (DSBs) were visualized within their chromatin context by labeling the Ku heterodimer. Single and clustered pKu70-labeled DSBs were quantified in euchromatic and heterochromatic regions at 0.1 h, 5 h and 24 h post-IR by transmission electron microscopy (TEM). Results: Increasing numbers of carbon ions per beam spot enhanced spatial clustering of DNA lesions and increased damage complexity with two or more DSBs in close proximity. This effect was detectable in euchromatin, but was much more pronounced in heterochromatin. Analyzing the dynamics of damage processing, our findings indicate that euchromatic DSBs were processed efficiently and repaired in a timely manner. In heterochromatin, by contrast, the number of clustered DSBs continuously increased further over the first hours following IR exposure, indicating the challenging task for the cell to process highly clustered DSBs appropriately. Conclusion: Increasing numbers of carbon ions applied to sub-nuclear chromatin regions enhanced the spatial clustering of DSBs and increased damage complexity, this being more pronounced in heterochromatic regions. Inefficient processing of clustered DSBs may explain the enhanced therapeutic efficacy of particle-based radiotherapy in cancer treatment.

## 1. Introduction

Radiotherapy is one of the major modalities for the efficient treatment of cancer. Tumor therapy with heavy ions is increasingly implemented, as dose distributions that are increasingly confined to the neoplasm and spare healthy tissues can be achieved [1]. Moreover, this dosimetric advantage is combined with the enhanced relative biological effectiveness (RBE) of this densely ionizing radiation (IR) [2]. Ionizing particles deposit concentrated ionization events along their trajectories, thereby presumably causing clustered DNA lesions [3,4]. Compared to conventional photon-based radiotherapy this unique energy deposition pattern of charged ions substantially improves therapeutic efficacy [5].

Repair of radiation-induced DSBs occurs within the higher-order chromatin structure [6]. The nuclear localization of DSBs can be related to the condensation status of chromatin, with the two extremes euchromatin versus heterochromatin [7]. Within the nucleus, chromatin is a highly dynamic structure and nucleosome modifications regulate DNA accessibility, flexibility and mobility for the recruitment of molecular machines ensuring transcription, replication and repair [8]. The chromatin state may influence the forms of radiation-induced DNA damage and may also affect repair processing [9]. Non-homologous end-joining (NHEJ) is widely considered as the prevalent DSB repair pathway, particularly in non-proliferating cells [10]. NHEJ mediates the fast ligation of broken DNA ends and is initiated by the binding of the Ku70/Ku80 heterodimer to DSB termini to generate the binding scaffold for other NHEJ factors [11].

Currently, different experimental techniques exist to visualize DSBs. Phosphorylated H2AX (γH2AX) and p53 binding protein 1 (53BP1) appear in foci of DNA damage under immunofluorescence microscopy (IFM) and are considered surrogate markers of DSBs [12]. However, the limited resolution of conventional light microscopy has restricted further insights into the detailed structure of repair foci. In recent years we established immunogold-labelling techniques to detect DNA-repair proteins within the chromatin ultrastructure using transmission electron microscopy (TEM) [13,14,15]. The nanometer resolution of TEM permits the visualization of repair proteins at single-molecule level in different chromatin compartments. By labeling phosphorylated Ku70 (pKu70, phosphorylated at serine 6) which binds directly to broken DNA ends in preparation for re-joining, this TEM approach permits the reliable detection of unrepaired DSBs in electron-lucent euchromatin and electron-dense heterochromatin [16]. In this study, we analyzed human fibroblasts, because the (epi)genome of these normal tissue cells is highly organized into chromosome territories, as well as into heterochromatin and euchromatin domains. The disruption of (epi)genomic integrity with the accumulation of various kinds of DNA damage due to deficient DNA repair capacities and epigenetic abnormalities constitute hallmarks of malignant tumors. Another important factor influencing radiation-induced DNA damage in tumor cells is the variable production of reactive oxygen species (ROS) as a consequence of hypoxic or metabolic stress. Due to these confounding factors in heterogeneous cancer cell populations we used human fibroblasts to analyze the fundamental chromatin-associated DNA repair mechanism.

The heavy-ion microbeam SNAKE (Superconducting Nanoprobe for Applied nuclear physics Experiments) enables the targeted irradiation of cell layers with defined numbers of carbon ions (1, 10 or 100 ions per point, ipp) focused to micrometer-sized stripes or spots [17]. The sub-micrometer beam size allows the targeted irradiation of cellular substructures with high targeting accuracy [18]. By scanning one beam stripe/spot after another, the heavy-ion microbeam can irradiate several cm^2^ of cell layers with pre-defined patterns. Using the focusing system of SNAKE, single and multiple carbon ions were delivered to micrometer-sized beam stripes/spots in targeted cell nuclei and at defined time-points after exposure DNA lesions were visualized by different high-resolution microscopy approaches. The objective of this investigation was to study the influence of high local damage density on the clustering of DSBs in different chromatin regions.

## 2. Results

### 2.1. IFM: γH2AX, 53BP1 and pKu70 Foci after Linear Microbeam IR

**Figure 1:**
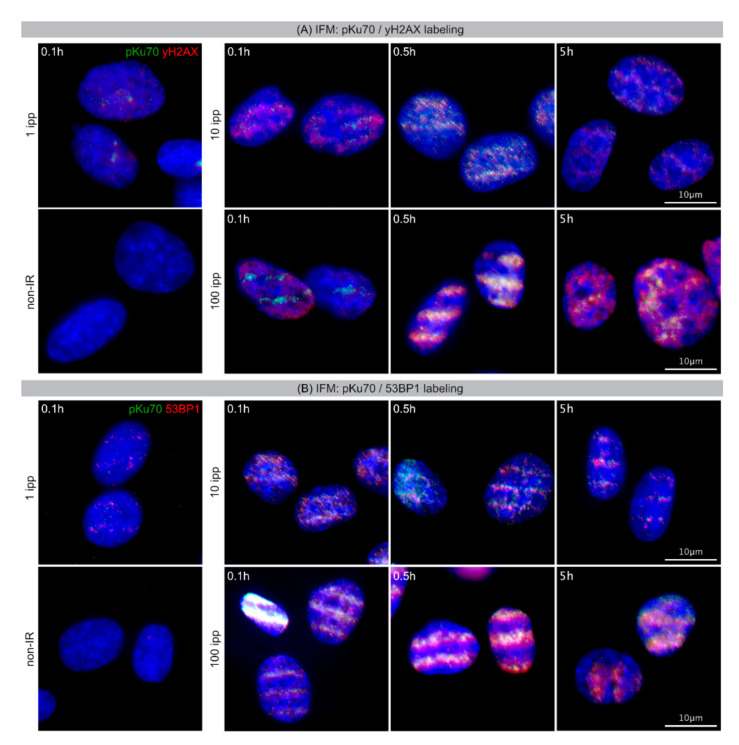
Radiation-induced DNA damage patterns visualized by conventional IFM. (**A**) Micrographs of double-staining for pKu70 (green) with γH2AX (red) in DAPI-stained nuclei analyzed at 0.1 h, 0.5 h and 5 h after linear microbeam IR with 1, 10 and 100 ipp carbon ions, compared to non-irradiated cells. (**B**) Micrographs of double-staining for pKu70 (green) with 53BP1 (red) in DAPI-stained nuclei analyzed at 0.1 h, 0.5 h and 5 h after linear microbeam IR with 1, 10 and 100 ipp carbon ions, compared to non-irradiated cells.

Using the ion microbeam SNAKE, fibroblasts grown as adherent monolayers on cell carrier foils were irradiated with defined numbers of carbon ions (1, 10 or 100 ipp) focused into micrometer-sized linear stripes. At 0.1 h, 0.5 h and 5 h post-IR cells were fixed and labeled with γH2AX and 53BP1 to visualize radiation-induced DNA damage foci by immunofluorescence microscopy (IFM). While non-irradiated cells revealed nearly no foci, weak signals of radiation-induced γH2AX and 53BP1 could be visualized at 0.1 h post-IR with 1 ipp (Figure 1). After microbeam IR with 10 or 100 ipp, linear arrangement of irregular-shaped, partially confluent γH2AX and 53BP1 signals at 0.1 h, 0.5 h and 5 h post-IR may indicate high levels of DSBs in these microbeam stripes. Focusing higher numbers of carbon ions to the micrometer-sized targets and thus creating a high local damage density, permits even the detection of the pKu70 protein that is recruited in low numbers to individual damage sites. As Ku-heterodimers bind directly to DNA double-stranded break ends, these discrete pKu70 signals at the center of more expanded 53BP1 signals may indicate unrepaired DSBs. Taking advantage of high-resolution STED microscopy, the fine structure of primary ion tracks was visualized at 0.1 h and 5 h after carbon ion IR. Using STED microscopy, the improved resolution revealed that primary ion trajectories were composed of small pKu70 subfoci in the middle of the damage trails (Supplementary Figure S1). Collectively, these fluorescent imaging microscopy approaches allow verification of successful sub-nuclear structure targeting, but do not enable precise characterization of radiation-induced DNA lesions in the chromatin environment.

### 2.2. TEM: Spatial Distribution of Radiation-Induced DNA Lesions after Linear Microbeam IR (10 ipp)

**Figure 2:**
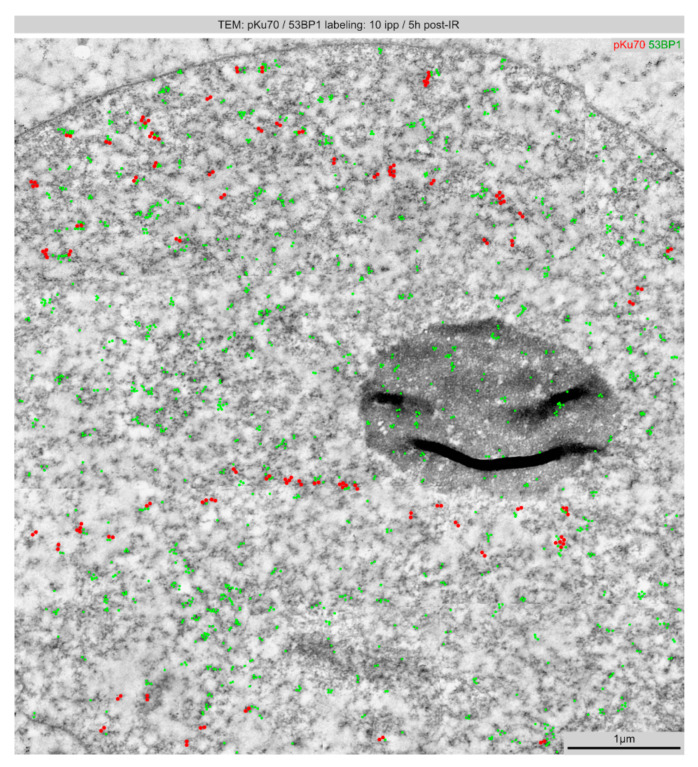
Spatial distribution of DNA lesions induced by multiple carbon ions (10 ipp) visualized by TEM. Micrograph of double-labeling for pKu70 (10 nm beads, colored in red) and 53BP1 (6 nm beads, colored in green) 5 h after microbeam IR with 10 ipp carbon ions.

TEM is becoming a valuable tool for studying the repair of DNA lesions in the context of chromatin. In previous TEM studies, we have shown that labeling the activated form of Ku70/Ku80 heterodimer permits detection of unrepaired DSBs (pKu70 dimers), whereas 53BP1 is diffusely recruited to damaged chromatin regions. Here, immunogold-labeling for pKu70 and 53BP1 and subsequent TEM imaging was used to visualize the ultrastructural pattern of DNA damage caused in cells exposed to ion beam IR. Irradiated cells were harvested as adherent monolayers to preserve their chromatin structure during sample processing. Linear microbeam IR with 10 ipp was performed to explore the radiation damage of multiple ion hits in micrometer-sized areas of the nucleus. TEM analysis with gold-labeling of pKu70 and 53BP1 revealed high densities of radiation-induced DNA lesions throughout the entire nuclear sections of irradiated cells (Figure 2). For improved visibility in the grey-scaled background of chromatin environment, the gold-beads (with varying sizes for different repair factors) were pseudo-colored to distinguish between pKu70 (10 nm gold-beads: red) and 53BP1 (6 nm gold-beads: green). Linear microbeam IR with 10 ipp produced microbeam stripes with increased proportions of clusters with multiple pKu70-dimers, which may reflect the high complexity of radiation-induced DNA damage (Figure 2). Due to the high damage levels, the linear microbeam IR was clearly traceable from the DNA damage pattern. Strikingly, we observed thousands of 53BP1 clusters scattered evenly throughout nuclear sections of all irradiated cells (Figure 2). Chromatin-binding protein 53BP1 regulates the repair of DSBs by suppressing nucleolytic resection of DNA termini [19]. Due to the lack of putative enzymatic activity, 53BP1 is supposed to serve as master scaffold to form functional complexes with DSB-responsive factors at damaged chromatin. While nuclear sections of non-irradiated fibroblasts revealed only single 53BP1 gold-beads, this global 53BP1-labeling may reflect extensive chromatin rearrangements during the repair process of clustered DNA damage. Moreover, by nanoscale resolution of TEM it becomes clear that the streak-shaped γH2AX and 53BP1 fluorescence signals observed by conventional microscopy consist of multiple separated clusters of DSBs within the particle trajectories.

### 2.3. TEM: Spatial Distribution of Radiation-Induced DNA Lesions after Focused Microbeam IR (1 ipp)

**Figure 3:**
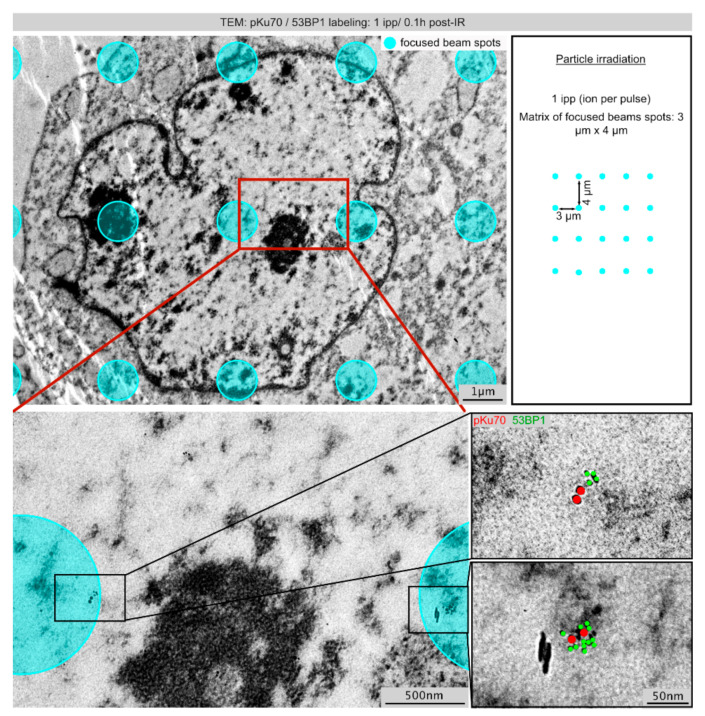
Spatial distribution of DNA lesions induced by single carbon ions (1 ipp) visualized by TEM. Micrograph of double-labeling for pKu70 (10 nm beads, colored in red) and 53BP1 (6 nm beads, colored in green) after focused microbeam IR with 1 ipp carbon ions. The pre-defined IR matrix with micrometer-sized beam spots is delineated as circular areas (cyan). Framed regions are shown at higher magnification.

Focused microbeam IR permits to expose subcellular targets to precise numbers of particles according to pre-defined grid matrix. For our matrix IR, beam spots (diameter ≈1 µm) were arranged in uniform 2D grids with 3 µm and 4 µm spacing for the x- and y-coordinates, respectively. After focused beam IR with 1 ipp, TEM imaging revealed single-ion hits with only small clusters of pKu70- and 53BP1-beads scattered sporadically throughout the nuclear sections (Figure 3). To match the verified DNA damage with the pre-defined IR grid matrix, presumed beam spots were plotted as blue dots (with diameters of ≈1 µm according to supposed beam precision) in TEM images of nuclear sections. Even 0.1 h post-IR heavy-ion induced DNA damage was detectable in only ≤50% of the targeted beam-spots. Carbon ions traversing the entire nucleus are supposed to generate high numbers of closely spaced DSBs along their trajectories. However, the lack of damage detection in most beam spots can be explained by the fact that only ultrathin nuclear sections were analyzed, suggesting that the low DNA damage level induced by single carbon ions cannot be detected on the respective nuclear plane. Single DSBs, comprising individual pKu70-dimers and multiple 53BP1 beads, were usually detectable at the border of compacted heterochromatin (Figure 3, lower panel).

### 2.4. TEM: Spatial Distribution of Radiation-Induced DNA Lesions after Focused Microbeam IR (10 ipp)

**Figure 4:**
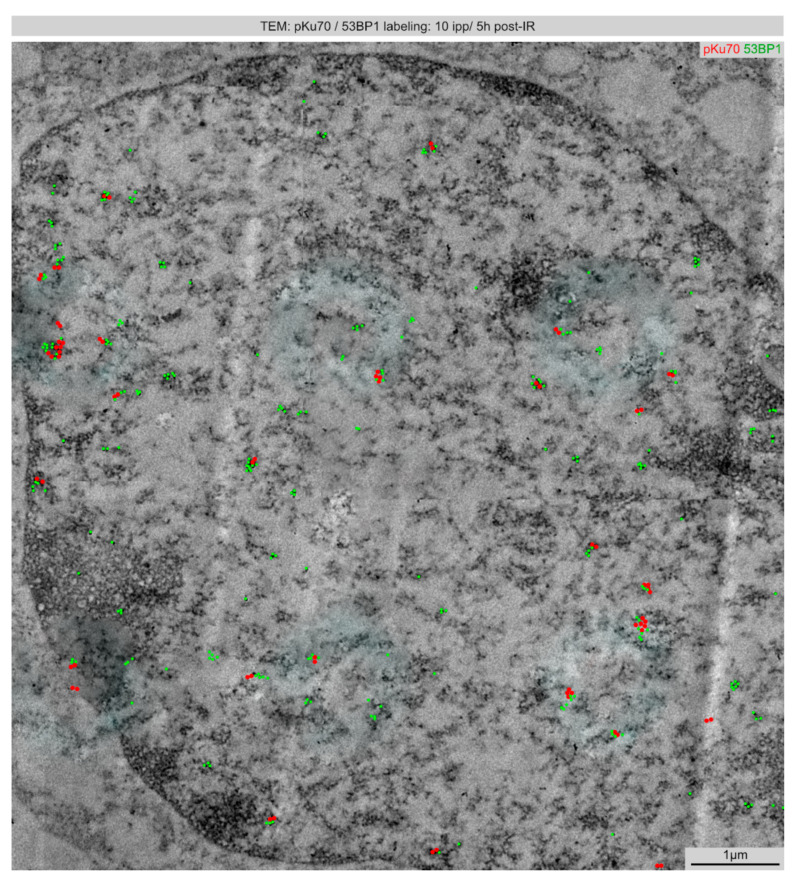
Spatial distribution of DNA lesions induced by multiple carbon ions (10 ipp) visualized by TEM. Micrograph of double-labeling for pKu70 (10 nm beads, colored in red) and 53BP1 (6 nm beads, colored in green) 5 h after focused microbeam IR with 10 ipp carbon ions.

Analyzing TEM sections after matrix IR with 10 ipp, multiple DNA lesions were observed in the nuclear sections, reflecting the higher number of applied carbon ions (Figure 4). However, the presumed beam spots from the pre-defined matrix IR cannot be anticipated from the detectable DNA damage sites (Figure 4). It cannot be excluded that targeting inaccuracies with some background IR or secondary particles are responsible for this finding. Moreover, the dynamic modifications of chromatin structures during the repair process may also lead to the relocation of damaged chromatin outside the defined beam spots. Confined movements of damaged chromatin may become necessary to allow access of condensed chromatin to the DNA repair machinery. Moreover, after microbeam IR with 10 ipp, more DNA damage sites with increased complexity (3–4 and ≥5 pKu70-beads) were measurable, particularly in heterochromatic regions (Figure 4). Collectively, our findings indicate that microbeam IR with multiple carbon ions focused to small nuclear volumes resulted in enhanced density and increased structural complexity of DNA lesions. However, limited accuracy of the beam line, emission of secondary electrons outside targeted volumes, or damage-induced remodeling of original chromatin structures may hamper to recognize the pre-defined matrix of focused microbeam IR.

Fibroblasts are characterized by a wide-ranging tolerance to ionizing irradiation and are able to handle hundreds of DSBs without loss of viability. By using very high doses focused to subnuclear regions (100 ipp: ≈442.2 Gy) the objective of this experiment was to investigate whether certain nuclear structures, such as specific chromatin compartments, are more vulnerable to radiation damage. However, due to the rather homogeneous chromatin architecture of human fibroblasts and their reduced cell survival after focused high-dose irradiation, it was not possible to answer this question in our experimental setting.

### 2.5. TEM: Quantification of Radiation-Induced pKu70 Clusters after Microbeam IR

**Figure 5:**
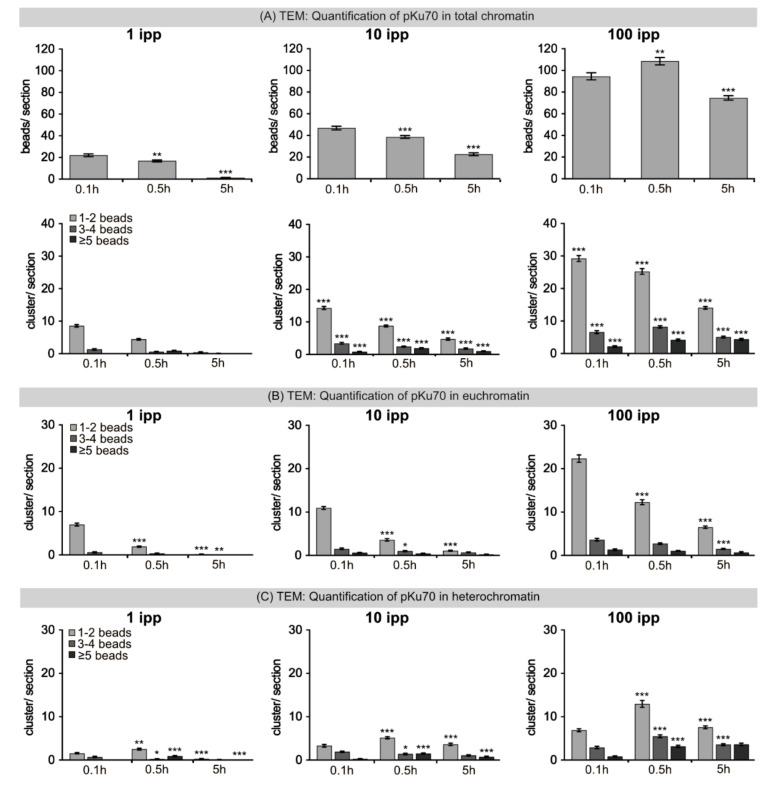
Quantification of pKu70 beads and clusters after microbeam IR with carbon ions. (**A**) For pKu70 the number of beads and clusters (with 1–2, 3–4 and ≥5 beads per cluster, reflecting lesion complexity) were quantified in the total chromatin of ≥25 nuclear sections after focused microbeam IR with 1, 10 and 100 ipp. Statistical significances for beads/section based on comparison to previous timepoint. Statistical significance for cluster/section based on comparison to equivalent cluster size and timepoint of lower dose (i.e., 10 ipp vs. 1 ipp, 100 ipp vs. 10 ipp). (**B**) For pKu70 the number of clusters (with 1–2, 3–4 and ≥5 beads per cluster) were shown in euchromatin after microbeam IR with 1, 10 and 100 ipp. Statistical significances based on comparison of equivalent cluster size and dose to previous timepoint. (**C**) For pKu70 the number of clusters (with 1–2, 3–4 and ≥5 beads per cluster) were shown in heterochromatin after microbeam IR with 1, 10 and 100 ipp. Statistical significances based on comparison of equivalent cluster size and dose to previous timepoint. Data are presented as mean of 3 technical replicates ± SEM. Significant statistical difference * (*p* <0.05), ** (*p* < 0.01), *** (*p* < 0.001).

To measure the yields of unrepaired DSBs after focused microbeam IR with defined traversals of carbon ions, the numbers of pKu70 beads and clusters were counted in euchromatic and heterochromatic regions of nuclear sections (Figure 5). Absolute measurements of pKu70 beads at 0.1 h after application of 1, 10 or 100 carbon ions revealed an approximate doubling of detectable radiation-induced DNA damage (1 ipp: 22.0 ± 1.3 beads/section; 10 ipp 46.9 ± 1.7 beads/section; 100 ipp: 94.6 ± 3.3 beads/section; Figure 5A). This lack of linear correlation between the number of applied ions and detectable DNA damage can be explained by the distinctly longer beam times with ongoing DNA repair. Following 1 ipp, the amount of radiation-induced DSBs significantly decreased to very low damage levels at 24 h post-IR (from 22.1 ± 1.3 to 1.2 ± 0.4 beads/section: decrease of 95%; Figure 5A). After 10 ipp radiation-induced DSBs were still efficiently repaired within 24 h post-IR (from 46.9 ± 1.7 to 22.6 ± 1.4 beads/section: decrease of ≈50%; Figure 5A). However, following 100 ipp, the extremely high damage levels increased even further from 0.1 h to 5 h post-IR (from 94.6 ± 3.3 to 108.5 ± 3.4 beads/section: increase to ≈115%; Figure 5A), suggesting that the repair machinery is overloaded and the NHEJ pathway may be compromised. To consider not only the number of DSBs but also their complexity, pKu70 clusters with 1–2 beads, 3–4 beads and ≥5 beads were counted separately in nuclear sections after exposure to 1, 10 and 100 ipp. After 1 ipp most radiation-induced DNA lesions represent simple DSBs (2 pKu70-beads) and the amount of clustered DSBs was extremely low (≤10%) (Figure 5A). After IR exposure with 10 ipp (≈30%) and particularly with 100 ipp (≈60%), the number of complex clusters with 3–4 and ≥5 pKu70 beads increased noticeably, due to the proximity of particle interactions (Figure 5A).

To analyze potential differences in terms of DSB complexity between those formed in euchromatin or heterochromatin, the pKu70 clusters of 1–2 beads, 3–4 beads and ≥5 beads were counted after IR exposure with 1, 10 and 100 ipp (Figure 5B,C). After 1 and 10 ipp most euchromatic lesions were single DSBs (1–2 beads), after 100 ipp the damage complexity slightly increased (3–4 and ≥5 pKu70 beads) (Figure 5B). In euchromatic regions the number of radiation-induced DNA lesions decreased with time, indicating an efficient DNA repair process (Figure 5B). In heterochromatin, increased levels of complex DNA lesions (3–4 or ≥5 pKu70-beads) were detectable, particularly with increasing numbers of particle traversals (Figure 5C). Following microbeam IR with 100 ipp, the numbers of complex lesions increased from 0.1 h to 5 h post-IR (for 1–2 pKu70 beads: from 6.8 ± 0.3 to 13.0 ± 0.8 cluster/section; for 3–4 pKu70 beads: from 2.9 ± 0.3 to 5.5 ± 0.3 cluster/section; for ≥5 pKu70 beads: 0.8 ± 0.1 to 3.2 ± 0.2 cluster/section; Figure 5C). Moreover, after exposure to multiple ion IR, the number of complex DNA lesions remained at high levels even 5 h post-IR, suggesting that clustered DNA damage was not efficiently repaired (Figure 5C). The distinctly more complex DNA damage induced by focused microbeam IR with multiple carbon ions may reflect severe processing problems during DNA repair. Collectively, our results indicate that both the original chromatin compaction and the density of ion particles determine the clustering of DSBs with increased complexity and these clustered and highly complex DSBs are repaired with slower kinetics or not at all.

## 3. Discussion

Particle radiotherapy is an important treatment modality for malignant tumors due to the favorable depth-dose profile and high relative biological effectiveness within the Bragg peak [1]. Treatment planning of particle radiotherapy relies on biophysical modelling to predict the biological effects induced by diverse types of ion beams [20]. These track-structure simulations enable the assessment of physical and chemical interactions of primary particles and their secondary electrons with the traversed medium. For a better understanding of the complex biological effects, however, these theoretical simulations need to be proven by experimental investigations in organic cells with physiological chromatin organization.

Previous reports using super-resolution fluorescence microscopy have shown the formation of clustered DNA repair foci along the particle tracks in heavy ion-irradiated cells [21,22]. Super-resolution fluorescence microscopy has managed to overcome the diffraction limit, thereby enabling the imaging of cellular structures with an improved resolution for many applications. At the same time, these super-resolution techniques retain the advantages of optical microscopy with regard to sample preservation, imaging flexibility and target specificity [23]. Although these advanced optical technologies do not reach the resolution values of electron microscopy (EM), they allow the extraction of quantitative information on spatial distributions of proteins or other macromolecules within subcellular compartments. An early and powerful approach to obtain detailed information at the nanoscale level is using electrons instead of photons. Following the same physical principal, but with much smaller wavelength, electron microscopy is able to achieve clearly higher resolving power. However, transmission and scanning EM techniques are technically demanding, relatively costly and time-consuming. Importantly, due to principles of signal detection, the possibilities to specifically label and visualize multiple cellular structures or components in one specimen is still limited. Moreover, chemical fixation and contrasting procedures and/or physical sectioning render specimens vulnerable to artifacts and exclude the option to observe living cells or organisms at high resolution in their unperturbed state. However, due the highest resolution power the TEM approach permits nanoscale visualization of clustered DSBs and characterization of lesion complexity in the local chromatin ultrastructure [13,14,15].

Accumulating experimental evidence indicates that the induction of radiation-induced damage to cellular DNA also depends on the local chromatin density [24]. Ion beams crossing different chromatin regions may influence the radiation-induced DNA damage pattern in human cell nuclei and may cause damage to variable degrees with different functional importance, from affecting single nucleosomes to entire chromatin fiber loops [25]. This work focused on the induction of DSBs and their clustering, as DSBs are commonly considered the most relevant DNA lesions for the biological effects of IR. The defining feature of DSBs is the complete disruption of the molecular continuity. By affecting both DNA strands, DSBs lack the possibility to use the complementary, undamaged strand as template to restore the sequence in the damaged strand. In this experimental study with human fibroblasts, radiation-induced DNA damage after microbeam IR was analyzed by fluorescence and electron microscopy to obtain information at different scales. While fluorescence microscopic analysis highlights the nuclear distribution of DNA damage, electron microscopic study provides high-resolution information on the spatial clustering of DNA lesions in different chromatin domains at the micro- to nanoscale level. Defined numbers of carbon ions were delivered in micrometer-sized beam spots, so that the quantitative effects of single and multiple ions regarding the induction of damage to cellular DNA and its repair by the cell can be studied in small sub-regions of the nucleus. During DSB formation, each broken terminus binds a Ku heterodimer and the two heterodimers then associate to form a bridging complex that recruits additional NHEJ proteins. By labeling the activated Ku heterodimer, we were able to capture some structural aspects of DSB lesions and to define different levels of DSB complexity (pKu70 clusters with 1–2, 3–4 or ≥5 beads). Multiple DSBs in close vicinity (≤10 nm distance) were scored as clustered lesions and the number of DSBs in clusters is referred to as DSB complexity. The quantification of pKu70 clusters in different chromatin compartments indicates that more severe forms of damage complexity (clusters with 3–4 and ≥5 beads) were generated in heterochromatic regions. Moreover, the number of clustered DSBs in heterochromatin increased with time, suggesting that the repair of these clustered DSBs was not only delayed, but actually inherently inefficient. Our findings suggest that the increased DSB complexity in heterochromatic domains may interfere with damage processing and likely increase the risk for errors.

Labeling the activated Ku heterodimer permits visualization of unrepaired DSBs in close proximity within their chromatin environment at the micro- to nanoscale level by high-resolution electron microscopy. In previous studies we established TEM-based techniques to investigate specific DNA repair factors in the chromatin ultrastructure by post-embedding immunogold-labeling procedures for fixed cell and tissue specimens [13,14,15]. The exploration of co-localization events in ultrathin sections is achieved by double-labeling with gold-conjugated secondary antibodies, characterized by differentially sized colloidal gold particles (6–10 nm in diameter). Scoring these gold-particles in electron-lucent and electron-dense regions of ultrathin nuclear sections permits quantification of specific repair factors in euchromatic and heterochromatic domains. Restriction endonucleases generate the simplest possible form of DSB, as they disrupt the continuity of the DNA molecule without chemically altering any of its constituent moieties. To verify our TEM-based detection technique for clustered DSBs, restriction endonuclease I-SceI was used to generate defined numbers of DSBs at specific sites of the genome [26]. Following fixation and embedding of these I-*Sce*I transfected cells, ultra-thin sections were prepared and genomically-integrated restriction sites were subsequently labeled for pKu70. Screening of these nuclear sections by TEM revealed clusters of pKu70 dimers, which bind proportionally to the restriction cuts located at specific nucleotide sequences within 440 base pairs (bp) in length (Supplementary Figure S2).

Due to varying electron absorption in euchromatin versus heterochromatin TEM permits to capture chromatin density distribution in the nucleus by different grey scales. In our TEM study gold-beads and clusters were counted and assigned to euchromatic or heterochromatic compartments based on their different chromatin densities; however, this approach do not reveal possible variations regarding structural organization. In previous experiments, we established gold-labeling of p53BP1 in combination with euchromatic or heterochromatic histone modifications (e.g., H3K9ac, H3K9me3) to analyze the impact of differently compacted chromatin on DNA repair [13,14,15]. The nuclear distribution of these histone modifications correlated clearly with chromatin density defined by different grey levels. Further characterization of these radiation-induced lesions by gold-labeling other histone modifications or repair factors would be beneficial, but these examinations would require much time and effort, especially in this experimental setting.

Collectively, our findings support the idea that the biological significance of clustered DSBs is attributed to the cells inability to process them efficiently. Among DNA lesions, clustered DSBs represent the highest level of overall damage complexity, likely adding substantial difficulty to processing attempts. Additionally, DSBs have the greatest probability of causing adverse biological effects, including cell death, mutation as well as malignant transformations [26]. The clustering of DSBs may, depending on overall organization, destabilize chromatin and jeopardize its potential to orchestrate efficient processing by NHEJ. Our experimental results improve the mechanistic knowledge of DSB clustering that underpin the biological effectiveness of particle irradiation and may have implications for radiotherapy with heavy ions as well as for radiation protection in space research.

## 4. Materials and Methods

Cell Culture: Human TERT fibroblasts (normal foreskin fibroblasts immortalized by ectopic expression of the catalytic subunit of the telomerase enzyme) were grown on cell carrier foils in Fibroblast Growth Medium (PromoCell) at 37 °C and 5% CO_2_. Confluent cell layers on carrier foils were used for experiments. Cells transfected with I-*Sce*I plasmid were kindly provided by G. Iliakis [26].

Ion irradiation: Carbon ion IR was performed at the Scanning Ion Microbeam SNAKE installed at the Munich 14 MV tandem accelerator. IR was performed by stripe/spot application of counted, individual carbon ions (1, 10, 100 ions per pulse, ipp) with initial beam energy in vacuum of 55 MeV. After leaving the beam transport vacuum through 7.5 µm Kapton foil, traversing an air gap (~30 µm) and cell carrier foil (~6 µm) the ions hit the cells. Due to energy loss in material the ions have an energy of 49.5 MeV and corresponding LET of 333 keV/µm. For matrix IR, beam spots (diameter ≈1 µm) were arranged in uniform 2D grids with 3µm and 4µm spacing for the x- and y-coordinates, respectively. Electrostatically scanning fields of 500 µm × 500 µm (for 1 and 10 ipp) or 300 µm × 300 µm (for 100 ipp) were irradiated with pre-defined matrices. In order to apply the right number of ions, PMT detectors were placed behind the samples enabling the counting of single ions. Once sufficient amounts of ions were applied the beam was stopped using fast electrostatic beam switch. The chosen IR geometry with defined ion numbers gives fluences F of 0.083 ions/µm^2^ for 1 ipp, 0.83 ions/µm^2^ for 10 ipp and 8.3 ions/µm^2^ for 100 ipp. By using the formula $D=\frac{F\times LET}{\rho }$ the dose D can be calculated using the density of water $\left(\rho =1\frac{g}{cm^{3}}\right)$ as approximation for cell density. This results in corresponding doses of 4.4 Gy, 44.2 Gy and 442.2 Gy, respectively.

For STED analysis non-focused small angle IR using SNAKE was used as described previously [2]. Cells were cultured on 22 × 22 mm^2^ coverslips and irradiated in air. During IR cells were covered with medium layers of ≈7.5 µm thickness. Energy loss led to final energy of 27 MeV and corresponding LET of 500 keV/µm. The center of the coverslips was irradiated with field sizes of 3.5 × 22 mm^2^ under an angle of 9° and lasted only few seconds. IR was performed with 0.03 ions/µm^2^ fluence and resulted in 2.4 Gy.

Immunofluorescence Microscopy: Cells on carrier foils were fixed in 4% paraformaldehyde and permeabilized in 0.2% Triton X-100. After blocking with 1% BSA in PBS overnight, cells were incubated with primary antibodies (γH2AX; 53BP1, Novus Biologicals, Wiesbaden Nordenstadt, Germany; pKu70, pSer5, Abcam, Cambridge, UK) followed by AlexaFluor-488 or AlexaFluor-568 secondary antibodies (Invitrogen, Karlsruhe, Germany). Finally, cells were mounted in VECTAshield^TM^ mounting medium with 4′,6-diamidino-2-phenylindole (DAPI, Vector Laboratories, Burlingame, CA, USA). Fluorescence images were captured using Nikon-Eclipse Ni fluorescence microscope equipped with charge-coupled-device camera and acquisition software (Nikon, Düsseldorf, Germany).

For STED microscopy cells were processed similarly and labeled with the same primary antibodies, but goat-anti-rabbit Abberior STAR 440SXP (Abberior Instruments, Göttingen, Germany) and goat-anti-mouse Chromeo 505 (Active Motif, La Hulpe, Belgium) were used as secondary antibodies. After embedding using ProlongGold antifade, cells were imaged using super-resolution optical CW STED microscope (Leica TCS SP 8 3X, Leica Microsystems, Wetzlar, Germany). Excitation laser wavelength of 470 nm was used for Abberior STAR 440SX and for Chromeo505 wavelength of 514 nm, both with power of ≈1 mW each. Detection ranges were 473–504 nm and 518–580 nm, respectively, with depletion laser at 592 nm (≈70 mW). STED laser was subdivided in lateral STED beam (40%) and axial STED beam (60% of the power).

Transmission Electron Microscopy: Cells were fixed with 2% paraformaldehyde and 0.05% glutaraldehyde in PBS. Fixed samples were dehydrated using increasing concentrations of ethanol and infiltrated with LR White resin overnight (Plano, Wetzlar, Germany). Subsequently, samples were embedded in fresh resin with accelerator at 37 °C until the resin polymerized. Ultrathin sections (≈70 nm) were prepared on Microtome Ultracut UCT (Leica, Wetzlar, Germany) with diamond knives (Diatome, Biel, Switzerland), gathered up on pioloform-coated nickel grids and processed for immunogold-labeling. Sections were placed on drops of blocking solution (Aurion, Wageningen, The Netherlands) to block non-specific staining. Afterwards sections were rinsed and incubated with primary antibodies (anti-53BP1, Novus Biologicals, Wiesbaden Nordenstadt, Germany; anti-pKu70, pSer5, Abcam, Cambridge, UK) overnight at 4 °C. After rinsing, secondary antibodies conjugated with 6 nm or 10 nm gold particles (Aurion, Wageningen, The Netherlands) were applied to the grids for 1 h. Sections were rinsed and fixed with 2% glutaraldehyde in PBS. All sections were stained with uranyl acetate and examined using Tecnai Biotwin™ transmission electron microscope (FEI, Eindhoven, The Netherlands). For quantification, single beads and bead clusters were counted in ≥25 randomly chosen nuclear sections. Detection, localization and quantification of gold beads and clusters were performed at the electron microscope by eye. Absolute numbers of gold particles were mapped over the entire nuclear compartment of 25 cross sections. Gold beads and clusters were counted and assigned to euchromatic or heterochromatic compartments based on chromatin density (defined by different gray scale levels). To facilitate the detection of beads and clusters in compact heterochromatin, regions of interest were overexposed by the electron beam to visualize electron-dense gold particles with their strong scattering. In TEM micrographs gold-particles were overlayed with colored dots.

Statistical analysis: Statistical analysis was performed using GraphPad Prism (San Diego, CA, USA) software. Data sets were analyzed for normal distribution using Shapiro–Wilk. Normally distributed data sets were subsequently analyzed using unpaired two-tailed Student’s *t*-test (equal variances under F-test) or Welch’s *t*-test (unequal variances under F-test). Non-parametric data were analyzed for statistical significance using two-tailed Mann–Whitney U test. A *p* < 0.05 was considered statistically significant, *p* < 0.01 as highly significant and *p* < 0.001 as extremely statistically significant.

## Data Availability

The data that support the findings of this study are available from the Department of Radiation Oncology, Saarland University Hospital, Homburg/Saar, Germany.

## Supplementary Materials

The following are available online at https://www.mdpi.com/article/10.3390/ijms22147638/s1, Supplementary Figure S1: STED micrographs show the fine structure of ion trajectories at 0.1 h and 5 h after carbon ion IR. Supplementary Figure S2: TEM micrograph of pKu70-labeled restriction cuts in I-SceI transfected cells.

## References

[1] Tinganelli W., Durante M. (2020). Carbon Ion Radiobiology. Cancers.

[2] Stewart R.D., Carlson D.J., Butkus M.P., Hawkins R., Friedrich T., Scholz M. (2018). A comparison of mechanism-inspired models for particle relative biological effectiveness (RBE). Med. Phys..

[3] Hagiwara Y., Oike T., Niimi A., Yamauchi M., Sato H., Limsirichaikul S., Held K.D., Nakano T., Shibata A. (2019). Clustered DNA double-strand break formation and the repair pathway following heavy-ion irradiation. J. Radiat. Res..

[4] Hagiwara Y., Niimi A., Isono M., Yamauchi M., Yasuhara T., Limsirichaikul S., Oike T., Sato H., Held K.D., Nakano T. (2017). 3D-structured illumination microscopy reveals clustered DNA double-strand break formation in widespread gammaH2AX foci after high LET heavy-ion particle radiation. Oncotarget.

[5] Malouff T.D., Mahajan A., Krishnan S., Beltran C., Seneviratne D.S., Trifiletti D.M. (2020). Carbon Ion Therapy: A Modern Review of an Emerging Technology. Front. Oncol..

[6] Stadler J., Richly H. (2017). Regulation of DNA Repair Mechanisms: How the Chromatin Environment Regulates the DNA Damage Response. Int. J. Mol. Sci..

[7] Kalousi A., Soutoglou E. (2016). Nuclear compartmentalization of DNA repair. Curr. Opin. Genet. Dev..

[8] Hauer M.H., Gasser S.M. (2017). Chromatin and nucleosome dynamics in DNA damage and repair. Genes Dev..

[9] Clouaire T., Legube G. (2019). A Snapshot on the Cis Chromatin Response to DNA Double-Strand Breaks. Trends Genet..

[10] Chang H.H.Y., Pannunzio N.R., Adachi N., Lieber M.R. (2017). Non-homologous DNA end joining and alternative pathways to double-strand break repair. Nat. Rev. Mol. Cell Biol..

[11] Frit P., Ropars V., Modesti M., Charbonnier J.B., Calsou P. (2019). Plugged into the Ku-DNA hub: The NHEJ network. Prog. Biophys. Mol. Biol..

[12] Bekker-Jensen S., Mailand N. (2010). Assembly and function of DNA double-strand break repair foci in mammalian cells. DNA Repair.

[13] Lorat Y., Brunner C.U., Schanz S., Jakob B., Taucher-Scholz G., Rube C.E. (2015). Nanoscale analysis of clustered DNA damage after high-LET irradiation by quantitative electron microscopy—the heavy burden to repair. DNA Repair (Amst).

[14] Lorat Y., Schanz S., Schuler N., Wennemuth G., Rube C., Rube C.E. (2012). Beyond repair foci: DNA double-strand break repair in euchromatic and heterochromatic compartments analyzed by transmission electron microscopy. PLoS ONE.

[15] Rube C.E., Lorat Y., Schuler N., Schanz S., Wennemuth G., Rube C. (2011). DNA repair in the context of chromatin: New molecular insights by the nanoscale detection of DNA repair complexes using transmission electron microscopy. DNA Repair (Amst).

[16] Lorat Y., Schanz S., Rube C.E. (2016). Ultrastructural Insights into the Biological Significance of Persisting DNA Damage Foci after Low Doses of Ionizing Radiation. Clin. Cancer Res. Off. J. Am. Assoc. Cancer Res..

[17] Drexler G.A., Siebenwirth C., Drexler S.E., Girst S., Greubel C., Dollinger G., Friedl A.A. (2015). Live cell imaging at the Munich ion microbeam SNAKE—A status report. Radiat. Oncol..

[18] Siebenwirth C., Greubel C., Drexler G.A., Reindl J., Walsh D.W.M., Schwarz B., Sammer M., Baur I., Pospiech H., Schmid T.E. (2019). Local inhibition of rRNA transcription without nucleolar segregation after targeted ion irradiation of the nucleolus. J. Cell Sci..

[19] Noordermeer S.M., Adam S., Setiaputra D., Barazas M., Pettitt S.J., Ling A.K., Olivieri M., Alvarez-Quilon A., Moatti N., Zimmermann M. (2018). The shieldin complex mediates 53BP1-dependent DNA repair. Nature.

[20] Friedland W., Schmitt E., Kundrat P., Baiocco G., Ottolenghi A. (2019). Track-structure simulations of energy deposition patterns to mitochondria and damage to their DNA. Int. J. Radiat. Biol..

[21] Hausmann M., Falk M., Neitzel C., Hofmann A., Biswas A., Gier T., Falkova I., Heermann D.W., Hildenbrand G. (2021). Elucidation of the Clustered Nano-Architecture of Radiation-Induced DNA Damage Sites and Surrounding Chromatin in Cancer Cells: A Single Molecule Localization Microscopy Approach. Int. J. Mol. Sci..

[22] Lopez Perez R., Best G., Nicolay N.H., Greubel C., Rossberger S., Reindl J., Dollinger G., Weber K.J., Cremer C., Huber P.E. (2016). Superresolution light microscopy shows nanostructure of carbon ion radiation-induced DNA double-strand break repair foci. FASEB J..

[23] Schermelleh L., Ferrand A., Huser T., Eggeling C., Sauer M., Biehlmaier O., Drummen G.P.C. (2019). Super-resolution microscopy demystified. Nat. Cell Biol..

[24] Hausmann M., Wagner E., Lee J.H., Schrock G., Schaufler W., Krufczik M., Papenfuss F., Port M., Bestvater F., Scherthan H. (2018). Super-resolution localization microscopy of radiation-induced histone H2AX-phosphorylation in relation to H3K9-trimethylation in HeLa cells. Nanoscale.

[25] Arnould C., Rocher V., Finoux A.L., Clouaire T., Li K., Zhou F., Caron P., Mangeot P.E., Ricci E.P., Mourad R. (2021). Loop extrusion as a mechanism for formation of DNA damage repair foci. Nature.

[26] Schipler A., Mladenova V., Soni A., Nikolov V., Saha J., Mladenov E., Iliakis G. (2016). Chromosome thripsis by DNA double strand break clusters causes enhanced cell lethality, chromosomal translocations and 53BP1-recruitment. Nucleic Acids Res..

