# Supplementary figures and images for: Focused Ion Microbeam Irradiation Induces Clustering of DNA Double-Strand Breaks in Heterochromatin Visualized by Nanoscale-Resolution Electron Microscopy
Source: Int J Mol Sci. 2021 Jul 16;22(14):7638. doi: 10.3390/ijms22147638 (PMC8306362; doi:10.3390/ijms22147638)

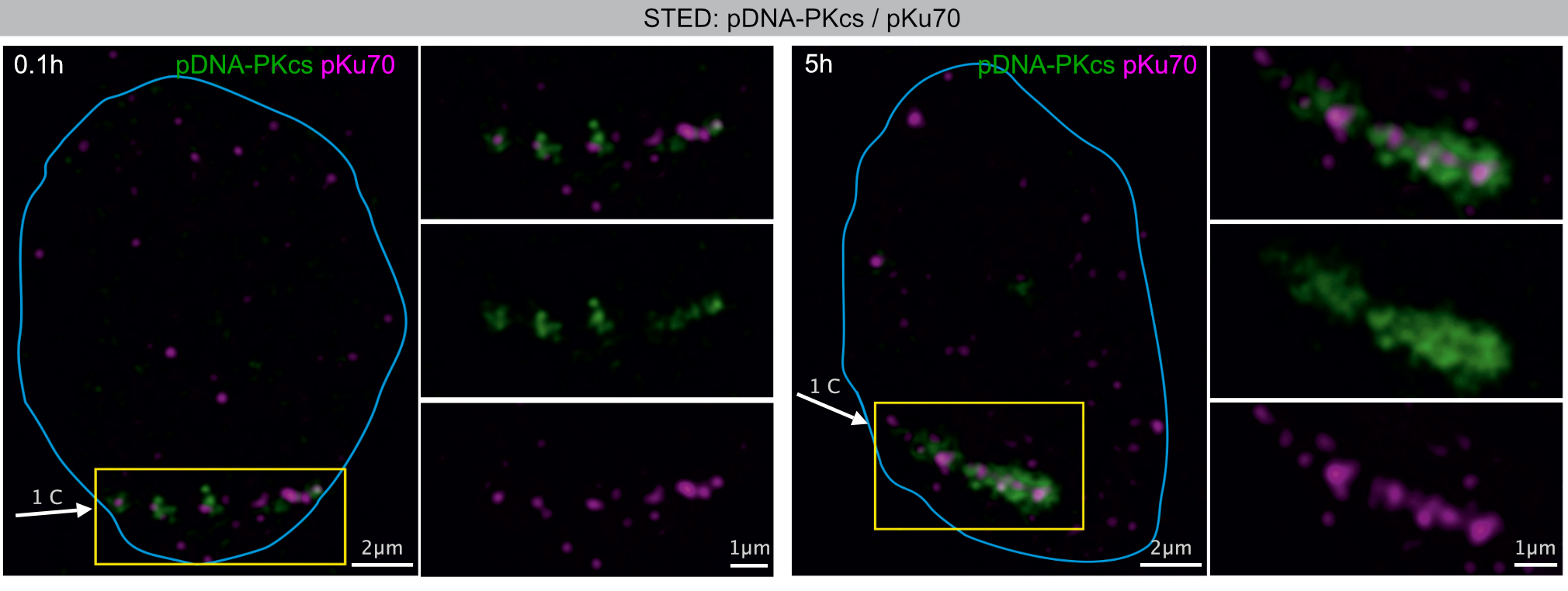

Supplement: Supplementary file 1 [file ijms-22-07638-s001.zip › Supplemental 1 v3.jpg]

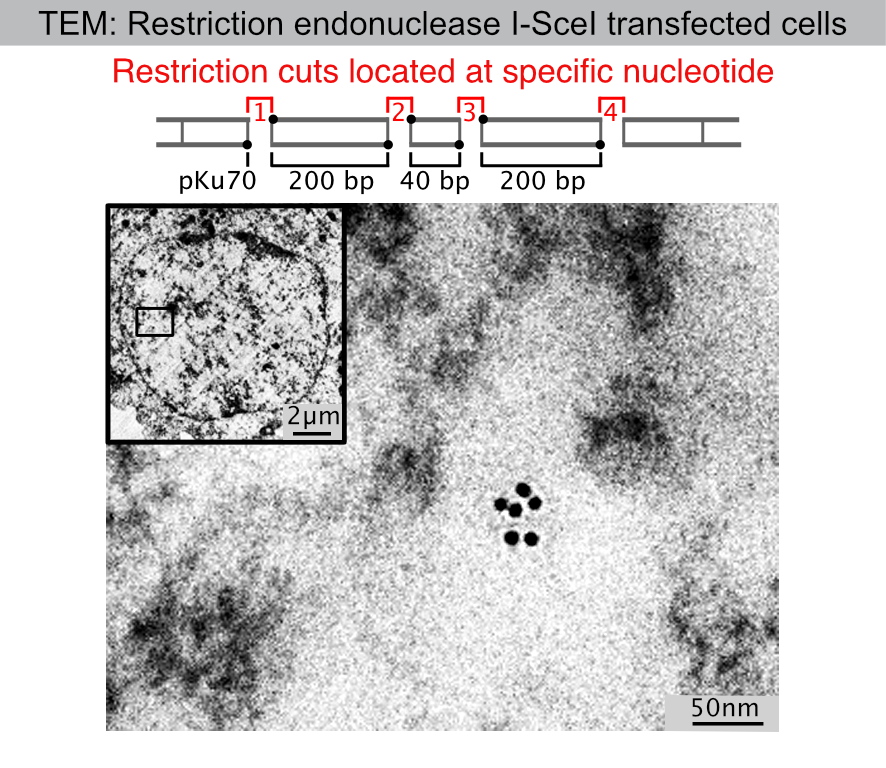

Supplement: Supplementary file 1 [file ijms-22-07638-s001.zip › Supplemental 2.tiff]
